# Melatoninergic Modulation of Benzene-Induced Pre-Leukemic Alterations in Rats: Effects of Melatonin, Agomelatine, and Luzindole: Short title: Melatoninergic Modulation in Benzene-Induced Pre-Leukemi

**DOI:** 10.31661/gmj.v15i.4245

**Published:** 2026-06-26

**Authors:** Mohammed M. Hussein M. Raouf, Ismail M Maulood, Zrar Saleem Marzany

**Affiliations:** ^1^ Department of Biology, Faculty of Science and Health, Koya University, Koya 44023, Kurdistan Region – F.R. Iraq; ^2^ Department of Biology, College of Science, Salahaddin University-Erbil, Kurdistan Region, Iraq; ^3^ Department of Biology, Faculty of Science and Health, Koya University, Koya 44023, Kurdistan Region – F.R. Iraq

**Keywords:** Benzene-Induced Pre-Leukemia, Melatonin, Agomelatine, Oxidative Stress, Bone Marrow Micronucleus, Activin A, Follistatin

## Abstract

**Background:**

Benzene is a well-established leukemogen associated with hematotoxicity, oxidative injury, inflammation, and genotoxic alterations, particularly in relation to acute myeloid leukemia (AML). Melatonin has antioxidant and anti-inflammatory properties that may exert protective effects against benzene-induced pre-leukemic alterations.

**Materials and Methods:**

This study evaluated melatoninergic modulation in a benzene-induced pre-leukemic rat model. Rats were assigned to five groups (n=8): control, benzene-only, benzene + melatonin (10 mg/kg), benzene + agomelatine (10 mg/kg), and benzene + melatonin + luzindole (0.2 mg/kg). Benzene was administered intravenously as a benzene:2-propanol: distilled water mixture (1:5:5, v/v/v) every 48 h for 4 weeks. Melatonin and agomelatine were administered orally once daily, while luzindole was used to assess melatonin receptor blockade. Hematological indices, leukocyte ratios, platelet parameters, bone marrow micronucleus endpoints, Activin A, Follistatin, MDA, 8-OHdG, CAT, and SOD were assessed.

**Results:**

Benzene exposure induced hematological disruption, altered leukocyte and platelet-related indices, increased micronucleus formation, and disturbed oxidative stress markers. Melatonin and agomelatine attenuated several benzene-induced alterations, including changes in erythropoietic indices, micronucleus frequency, leukocyte distribution, platelet-related parameters, MDA, and CAT activity. Luzindole partially reduced selected protective effects, suggesting that some responses may involve melatonin receptor-related pathways. However, inconsistent Activin A trends and unchanged Follistatin levels require cautious interpretation.

**Conclusion:**

Melatonin and agomelatine attenuated several hematological, genotoxic, and oxidative alterations in benzene-exposed rats. Luzindole partially modified these effects, suggesting possible melatonin receptor involvement alongside non-receptor antioxidant and anti-inflammatory actions.

## Introduction

Benzene is a recognized environmental and occupational leukemogen that primarily affects the hematopoietic system. Chronic or repeated benzene exposure has been associated with bone marrow suppression, oxidative stress, immune dysregulation, chromosomal instability, and an increased risk of hematological malignancies, particularly acute myeloid leukemia (AML) [1, 2]. In experimental studies, benzene exposure is commonly used to reproduce early hematotoxic and genotoxic alterations that resemble pre-leukemic changes, including leukocyte imbalance, erythropoietic suppression, micronucleus formation, oxidative injury, and disruption of bone marrow function [3, 4].

In the present study, the term pre-leukemia refers to an early benzene-induced pathological state characterized by hematological, cytological, and genotoxic abnormalities before the development of overt leukemia [5]. This classification was based on measurable experimental endpoints, including altered WBC and differential leukocyte counts, changes in erythropoietic indices such as PCE, NCE, and PCE/NCE ratio, increased MN-PCE and MN-NCE frequencies, oxidative stress biomarker disturbance, and platelet-related inflammatory indices. These parameters provide a practical experimental basis for identifying early benzene-induced hematopoietic injury and for evaluating potential protective interventions [5, 6].

Oxidative stress is considered one of the major mechanisms involved in benzene-induced hematotoxicity. Benzene metabolites can promote ROS generation, lipid peroxidation, DNA oxidative injury, and impairment of antioxidant defenses [2, 7]. Biomarkers such as MDA, 8-OHdG, CAT, and SOD are therefore useful for assessing oxidative damage and antioxidant response in benzene-exposed models [8, 9]. In parallel, bone marrow micronucleus endpoints, including MN-PCE and MN-NCE, are widely used to evaluate chromosomal damage and genotoxicity, while PCE and NCE proportions reflect erythropoietic activity and marrow toxicity [5].

Melatonin is a neurohormone involved in circadian rhythm regulation and is also recognized for its antioxidant, anti-inflammatory, immunomodulatory, and cytoprotective properties [10, 11, 12]. These effects may occur through both receptor-dependent and receptor-independent mechanisms. The membrane receptors MT1 and MT2 are involved in several cellular responses, including regulation of oxidative stress, inflammation, apoptosis, and immune signaling [13, 14]. However, melatonin can also act directly as a free-radical scavenger and may influence antioxidant defense independently of receptor activation [15, 16].

Agomelatine is a melatoninergic receptor agonist with activity at MT1 and MT2 receptors, whereas luzindole is commonly used as a melatonin receptor antagonist in experimental studies [17, 18]. Using these pharmacological modulators may help clarify whether selected protective responses are related to melatonin receptor signaling. However, interpretation must remain cautious because agomelatine may have additional pharmacological actions, and the absence of all possible antagonist-combination groups limits definitive mechanistic conclusions.

Activin A and Follistatin are involved in inflammation, hematopoietic regulation, and tissue response to injury. Their imbalance has been linked to inflammatory and neoplastic diseases, including hematological disorders [19, 20, 21]. However, alterations of this axis are modulated by the disease stage, tissue compartment, timing of measurement, and regulatory feedback mechanisms. Therefore, Activin A and Follistatin should be interpreted as supportive biomarkers rather than as direct proof of endocrine regulation in benzene-induced pre-leukemic alterations. Against this background, the present study aimed to evaluate the effects of melatonin, agomelatine, and luzindole on hematological changes as well as leukocyte counts and ratios, platelet indices, bone marrow micronucleus endpoints, oxidative stress biomarkers, and the Activin A/Follistatin axis in a benzene-induced pre-leukemic rat model. The study also examined whether selected protective effects could be linked to melatonin receptor-related mechanisms while recognizing the limitations of pharmacological inference in this experimental design.

## Materials and Methods

### Experimental Animals and Housing

This study used eight- to ten-week-old rats weighing 200–220 g. All animals were maintained under standard laboratory conditions with a 12:12 h light–dark cycle, controlled room temperature, and free access to standard chow and water. All rats were allowed to acclimatize before the experimental procedures. Animals were monitored daily for general health status, activity, grooming behavior, posture, respiratory pattern, and signs of distress. Environmental enrichment was provided, and humane endpoints were predefined to minimize animal suffering. The study protocol was approved by the Cihan University-Erbil Ethics Committee (Approval Number: CUE-REC/2025/06). All procedures were conducted according to institutional animal welfare standards and international guidelines for the care and use of laboratory animals. The study was reported in accordance with ARRIVE recommendations. Animals were randomly assigned to experimental groups before treatment initiation. No animals were excluded from the final analysis unless exclusion criteria were based on predefined welfare or technical criteria.

### Preparation of Dosing Solutions

Benzene was obtained from Scharlau (Scharlab S.L., Barcelona, Spain). The benzene dosing solution was prepared by mixing benzene, 2-propanol, and distilled water at a 1:5:5 (v/v/v) ratio in sealed amber glassware. The mixture was vortexed until a clear and homogeneous solution was obtained. All benzene-related procedures were conducted inside a certified fume hood to minimize volatilization and operator exposure. Melatonin (Bioven Ingredients, India) and agomelatine (Mylan, Germany) were freshly prepared on each dosing day. The required amount of each compound was calculated according to the most recent body weight of each animal and dissolved in distilled water to obtain a dose of 10 mg/kg. The preparations were protected from light using amber containers and mixed immediately before administration. Luzindole (Solarbio, China) was prepared at a dose of 0.2 mg/kg. The required amount was first dissolved in the minimum possible volume of absolute ethanol and then diluted with distilled water to obtain the final dosing preparation. The final ethanol concentration was kept as low as possible and was matched across relevant vehicle preparations when applicable. All formulations were prepared under clean laboratory conditions, labeled with the compound name and preparation time, protected from light when required, and used on the same day of preparation.

### Induction of the Benzene-Induced Pre-Leukemic Model

A benzene-induced pre-leukemic state was established through repeated intravenous administration of the benzene dosing solution. The formulation consisted of benzene, 2-propanol, and distilled water at a 1:5:5 (v/v/v) ratio. Each rat received 0.2 mL of the prepared solution, equivalent to approximately 15.9 mg of benzene per administration. Based on the animals’ body weights, the administered benzene dose was approximately 72–80 mg/kg per administration. The solution was administered intravenously through the tail vein every 48 hours for 4 weeks. The dose was calculated according to the individual body weight of each animal before administration. In the present study, the term pre-leukemic state refers to early hematological, cytological, and genotoxic alterations induced by repeated benzene exposure before the development of overt leukemia. This classification was based on measurable experimental criteria, including changes in WBC and differential leukocyte counts, altered erythropoietic indices, increased MN-PCE and MN-NCE frequencies, disturbance of oxidative stress biomarkers, and platelet-related inflammatory indices. These endpoints were used to evaluate early benzene-induced hematopoietic injury and treatment-related protective effects.

### Experimental Design and Treatment Regimens

Rats were randomly allocated into five groups, with eight animals in each group (n=8). The sample size was determined based on a previous experimental study of benzene-induced hematotoxicity in rats, which used eight animals per group and demonstrated significant differences in hematological and oxidative stress outcomes [3, 4]. Accordingly, eight rats were included in each experimental group to provide adequate statistical power while minimizing animal use. The experimental groups were arranged as follows:

Group 1 (Control): rats received distilled water by oral gavage once daily for 4 weeks.

Group 2 (Benzene): rats received 0.2 mL of the benzene dosing solution through the tail vein every 48 h for 4 weeks.

Group 3 (Benzene + Melatonin): rats received the same benzene regimen as Group 2 and were treated with melatonin at a dose of 10 mg/kg/day by oral gavage for 4 weeks.

Group 4 (Benzene + Agomelatine): rats received the same benzene regimen as Group 2 and were treated with agomelatine at a dose of 10 mg/kg/day by oral gavage for 4 weeks.

Group 5 (Benzene + Melatonin + Luzindole): rats received the same benzene regimen as Group 2 and were treated with melatonin at a dose of 10 mg/kg/day by oral gavage. Luzindole was administered at a dose of 0.2 mg/kg/day by oral gavage 15 min before melatonin administration for 4 weeks.

The luzindole group was included to evaluate whether selected protective effects of melatonin were modified by melatonin receptor blockade. The study did not include a benzene + agomelatine + luzindole group; therefore, antagonist-related findings were interpreted specifically in relation to melatonin + luzindole treatment and not as direct evidence that luzindole blocked agomelatine effects. Accordingly, receptor-mediated conclusions were interpreted cautiously and were limited to the experimental comparisons available in this design. All doses were adjusted according to body weight. Treatments were administered at a consistent time of day to reduce circadian and handling-related variation. Vehicle exposure was kept consistent as far as technically possible across treatment groups. All orally administered treatments were delivered by oral gavage at a standardized volume adjusted according to the body weight of each animal.

### Bone Marrow Smear Preparation and Romanowsky Staining

The supernatant was discarded, and the cell pellet was resuspended in PBS to obtain a homogeneous bone marrow cell suspension. At least five smears were prepared for each animal using the wedge-smear technique on clean, labeled glass slides at an approximate angle of 30°-45° to obtain a thin monolayer with a feathered edge. Smears were air-dried, fixed in absolute methanol, and stained sequentially with May-Grünwald and Giemsa stains. After staining, the slides were gently rinsed with buffered water and air-dried in a dust-free area. Smear quality was verified before microscopic examination to confirm uniform thickness, intact cellular morphology, and the absence of marked clumping or artifacts. The slides were screened using bright-field microscopy, and a comprehensive cytomorphological assessment was performed under oil immersion at 1000× magnification. The standard procedure was performed with minor laboratory modifications (Asita & Molise, 2011).

### Bone Marrow Micronucleus Assay

Genotoxicity and erythropoietic activity were evaluated using the bone marrow micronucleus assay. Bone marrow smears were prepared, fixed, and stained as described above. Slides were coded before microscopic examination to reduce observer bias. Micronuclei were identified according to standard morphological criteria: a micronucleus was recorded as a round or oval, distinctly stained, non-refractile body clearly separated from the main nucleus, free of staining artifacts, and not larger than one-third of the main nuclear diameter. For each animal, at least 1000 PCEs were examined to determine MN-PCE frequency. When required, 1000 NCEs were also evaluated to calculate MN-NCE frequency. Erythropoietic activity was expressed using the following indices:

PCE%=PCE/(PCE + NCE) × 100

NCE%=NCE/(PCE + NCE) × 100 

PCE/NCE ratio=PCE/NCE

Microscopic assessment was performed under oil immersion at 1000× magnification. Borderline or unclear fields were rechecked, and duplicate slides were reviewed when necessary. Slides were interpreted by investigators blinded to the experimental group allocation. Peripheral Blood Smear At the end of the experiment, peripheral blood samples were collected into EDTA-containing tubes. Thin blood films were prepared immediately on clean, labeled glass slides, air-dried, fixed in absolute methanol, and stained using May-Grünwald and Giemsa according to the Romanowsky staining method. Blood smears were examined using bright-field microscopy under oil immersion at 1000× magnification. Differential leukocyte counts were performed by counting 100 consecutive leukocytes per smear. Blast-like cells were identified based on standard cytomorphological features, including a high nuclear-to-cytoplasmic ratio, fine chromatin, prominent nucleoli, and basophilic cytoplasm. To reduce observer bias, slides were anonymized and examined independently by two investigators. Discordant findings were reviewed jointly until consensus was reached.

### Complete Blood Count

Blood samples were analyzed using an automated hematology analyzer (Mindray BC-2800Vet, Mindray Bio-Medical Electronics Co., Ltd., Shenzhen, China) for CBC assessment. The evaluated parameters included WBC, NEU, LYM, MON, EOS, BAS, PCV, MCV, RDW, MCH, MCHC, PLT, MPV, PDW, PCT, PLCR, and PLCC. Leukocyte differential counts were reported as absolute values and/or percentages according to the analyzer output. The derived inflammatory and hematological ratios were calculated as follows: LYM/MON, NEU/LYM, NEU/MON, MPV/PLT, PLT/LYM, WBC/MPV, RDW/PLT, and SII. Analyzer calibration and internal quality-control procedures were performed according to the manufacturer’s instructions. Smear verification was performed when abnormal or flagged results were detected. Measurement of Antioxidant Enzymes and Oxidative Stress Biomarkers SOD, CAT, and 8-OHdG were quantified using commercial rat ELISA kits from Sunlong Biotech Co., Ltd. (Hangzhou, Zhejiang Province, China), according to the manufacturer’s instructions. The catalog numbers were as follows: Rat Superoxide Dismutase (SOD) ELISA Kit, SL0664Ra; Rat Catalase (CAT) ELISA Kit, SL1084Ra; and Quick Step Rat 8-hydroxy-2′-deoxyguanosine (8-OHdG) ELISA Kit, QS0019Ra. Serum Activin A and Follistatin concentrations were measured using commercial rat-specific ELISA kits obtained from Sunlong Biotech Co., Ltd. (Hangzhou, Zhejiang, China; Activin A; Follistatin), according to the manufacturer’s instructions.. Samples were measured in duplicate, and standard curves were generated before calculating biomarker concentrations. Optical density was measured at 450 nm using a microplate reader (BioTek ELx800, BioTek Instruments Inc., Winooski, VT, USA). MDA was measured as an index of lipid peroxidation using the TBA reaction. Briefly, 150 µL of serum was deproteinized with TCA, reacted with TBA, and incubated at 95 °C for 45 min. After cooling and centrifugation, the absorbance of the MDA-TBA adduct was measured at 532 nm using a UV-visible spectrophotometer (Shimadzu UV-1800, Shimadzu Corporation, Kyoto, Japan) (23).

### Statistical Analysis

Statistical analysis was performed using GraphPad Prism version 9.0 (GraphPad Software, San Diego, CA, USA). Data were expressed as mean ± standard error (SE). Normality was assessed using the Shapiro–Wilk test, and homogeneity of variance was evaluated using the Brown–Forsythe or Levene’s test, as appropriate. Comparisons among groups were performed using ordinary one-way ANOVA. When the overall ANOVA showed a statistically significant difference, Tukey’s post hoc multiple-comparison test was used for pairwise group comparisons. The main comparisons included benzene versus control, treatment groups versus benzene, and luzindole co-treatment versus melatonin-treated rats where applicable. No data were excluded from the final analysis unless exclusion was required according to predefined technical or welfare-related criteria. CBC and biochemical biomarker data were analyzed using coded sample identifiers whenever applicable to reduce analytical bias. Outliers, if detected, were checked for technical or recording errors before analysis. A two-tailed P<0.05 was considered statistically significant. Exact P values were reported where available from GraphPad Prism output. Where exact P values were not available, threshold-based significance levels were reported as P<0.05, P<0.01, P<0.001, or P<0.0001, and the corresponding significance symbols were explained in the relevant figure legends and table footnotes.

## Results

**Figure-1:**
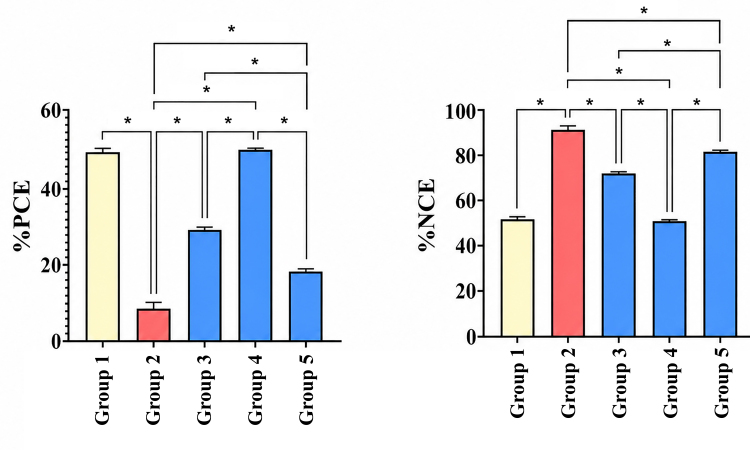
Figure 1. Effects of melatonin, agomelatine, and luzindole on %PCE and %NCE in benzene-exposed rats. Data are presented as mean ± SE (n=8 per group). Group 1:Control; Group 2: Benzene; Group 3: Benzene + Melatonin; Group 4: Benzene + Agomelatine; Group 5: Benzene + Melatonin + Luzindole. PCE: polychromatic erythrocyte; NCE: normochromatic erythrocyte; MEL: melatonin; Ago-MEL, agomelatine. *P<0.0001 indicate significant differences.

**Figure-2:**
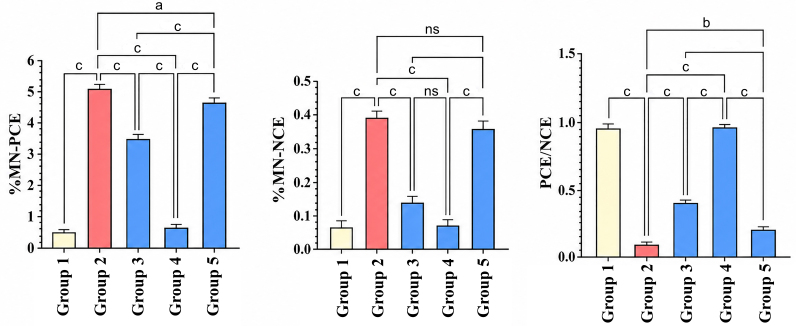
Figure 2. Effects of melatonin, agomelatine, and luzindole on %MN-PCE, %MN-NCE, and PCE/NCE ratio in benzene-exposed rats. Data are presented as mean ± SE (n=8 per group). Group 1:Control; Group 2: Benzene; Group 3: Benzene + Melatonin; Group 4: Benzene + Agomelatine; Group 5: Benzene + Melatonin + Luzindole. MN-PCE: micronucleated polychromatic erythrocyte; MN-NCE: micronucleated normochromatic erythrocyte; PCE: polychromatic erythrocyte; NCE: normochromatic erythrocyte; MEL: melatonin; Ago-ME: agomelatine. aP<0.05, bP<0.01, and cP<0.0001 indicate significant differences.

**Figure-3:**
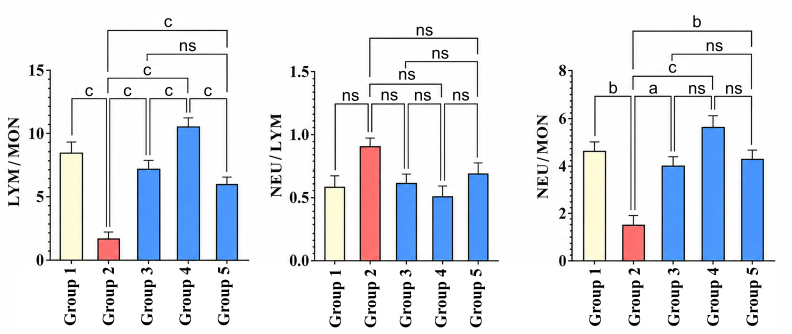
Figure 3. Effects of melatonin, agomelatine, and luzindole on leukocyte ratios in benzene-exposed rats. Data are presented as mean ± SE (n=8 per group). Group 1:Control; Group 2: Benzene; Group 3: Benzene + Melatonin; Group 4: Benzene + Agomelatine; Group 5: Benzene + Melatonin + Luzindole. LYM/MON: lymphocyte-to-monocyte ratio; NEU/LYM: neutrophil-to-lymphocyte ratio; NEU/MON: neutrophil-to-monocyte ratio; MEL: melatonin; Ago-MEL: agomelatine. aP<0.05, bP<0.01, and cP<0.0001 indicate significant differences. ns: Not Significant.

**Figure-4:**
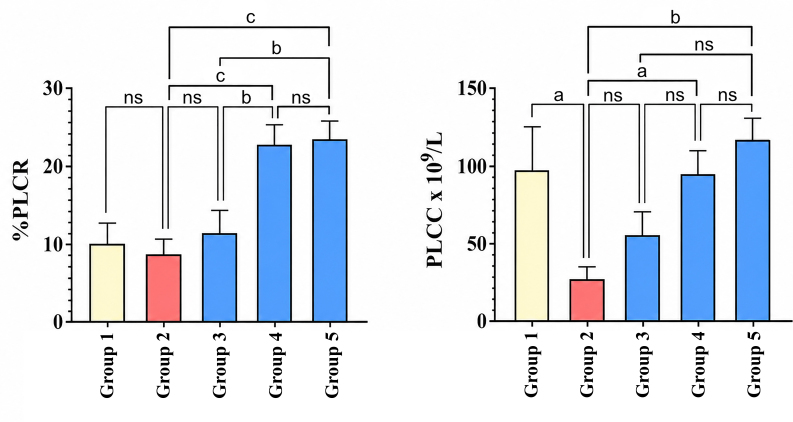
Figure 4. Effect of melatonin, agomelatine, and luzindole on %PLCR and PLCC in benzene-exposed rats. Data are presented as mean ± SE (n=8 per group). Group 1:Control; Group 2: Benzene; Group 3: Benzene + Melatonin; Group 4: Benzene + Agomelatine; Group 5: Benzene + Melatonin + Luzindole. PLCR: platelet large cell ratio; PLCC: platelet large cell count. aP<0.05, bP< 0.01, and cP<0.001 indicate significant differences. ns: Not Significant.

**Figure-5:**
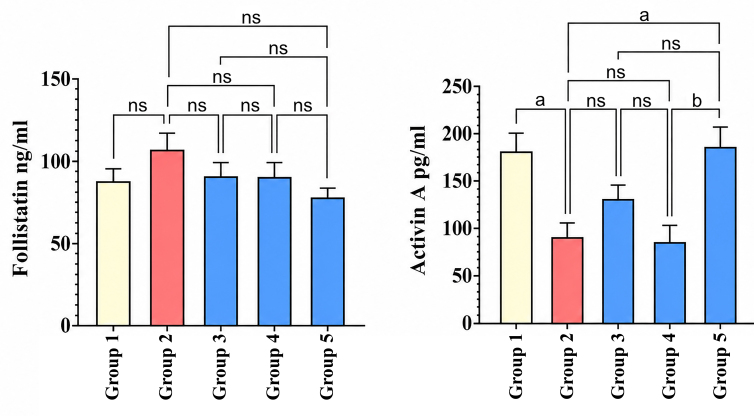
Figure 5. Effects of melatonin, agomelatine, and luzindole on Activin A and Follistatin levels in benzene-exposed rats. Data are presented as mean ± SE (n=8 per group). Group 1:Control; Group 2: Benzene; Group 3: Benzene + Melatonin; Group 4: Benzene + Agomelatine; Group 5: Benzene + Melatonin + Luzindole. aP<0.05 ,and bP<0.01 indicate significant differences. ns: Not Significant.

**Figure-6:**
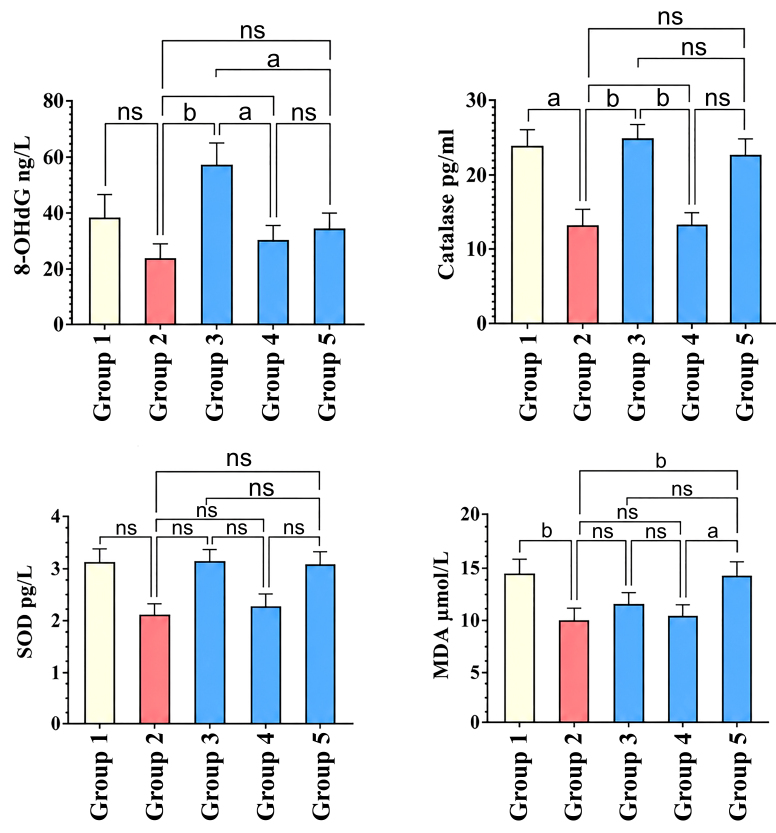
Figure 6. Effects of melatonin, agomelatine, and luzindole on oxidative stress and antioxidant biomarkers in benzene-exposed rats. Data are presented as mean ± SE (n=8 per group). Group 1:Control; Group 2: Benzene; Group 3: Benzene + Melatonin; Group 4: Benzene + Agomelatine; Group 5: Benzene + Melatonin + Luzindole. 8-OHdG: 8-hydroxy-2′-deoxyguanosine; CAT: catalase; SOD: superoxide dismutase; MDA: malondialdehyde; MEL: melatonin; Ago-MEL: agomelatine. aP<0.05,and bP<0.01 indicate significant differences. ns: Not Significant.

**Table:** Table1. Effects of Melatonin, Agomelatine, and Luzindole on Differential Leukocyte Counts in Benzene-Exposed Rats

Parameters	Group 1	Group 2	Group 3	Group 4	Group 5
WBC count (cells ×10³/µL)	11.93± 0.99a	7.53 ± 0.72	9.13± 0.99	9.901 ± 1.128	10.59 ± 0.7576
NEU count (cells ×10³/µL)	3.07± 0.31	4.04± 0.7	2.65± 0.33	2.984 ± 0.4693	4.056 ± 0.3041
LYM count (cells ×10³/µL)	7.936 ±0.8	5.53± 0.54	5.03± 0.63	6.00 ± 0.00	5.90 ± 0.00
MON count (cells ×10³/µL)	0.8± 0.19c	0.41 ± 0.05c	3.46 ± 0.48c	0.55 ± 0.00c	0.94 ± 0.00c
EOS count (cells ×10³/µL)	0.1± 0.03b	0.13 ± 0.04	0.2 ± 0.03‡	0.2000 ±0.03273	0.2000 ± 0.03273
BAS count (cells ×10³/µL)	0.01± 0.008b	0.12± 0.04	0.15± 0.04†	0.2± 0.03273	0.2000 ± 0.03273

Values are presented as mean ± SE. Group 1:Control; Group 2: Benzene; Group 3: Benzene + Melatonin; Group 4: Benzene + Agomelatine; Group 5: Benzene + Melatonin + Luzindole. WBC: white blood cell; NEU: neutrophil; LYM: lymphocyte; MON, monocyte; EOS, eosinophil; BAS, basophil; . aP<0.05, bP<0.01,and cP<0.001 indicate significant differences compared with the benzene group; †P<0.05 and ‡P<0.001 indicate significant differences compared with the benzene + melatonin group.

**Table:** Table2. Effects of Melatonin, Agomelatine, and Luzindole on Platelet Indices in Benzene- Exposed Rats

Parameters	Group 1	Group 2	Group 3	Group 4	Group 5
PLT count (cells ×10³/µL)	435.6 ± 41.51	424.6 ± 61.74	388.6 ± 67.59	451.4 ± 61.26	564.9 ± 89.9
MPV count (fL)	9.384 ± 0.26	6.47 ± 0.08	6.86 ± 0.21	7.650 ± 0.32	7.55 ± 0.22
PDW count (fL)	12.53 ± 0.60‡	9.05 ± 0.41	9.123 ± 0.32	9.16 ± 1.17	9.98 ± 1.48
%PCT	0.4 ± 0.04	0.27 ± 0.03	0.25 ± 0.04*	0.33± 0.04	0.4 ± 0.02
MPV/PLT	0.02 ± 0.003	0.01 ± 0.003	0.02 ± 0.01	0.03 ± 0.01	0.03 ± 0.02
PLT/LYM	59.24 ± 7.68	76.47 ± 8.27	81.69 ± 17.78	75.23 ± 10.21	95.74 ± 15.24
WBC/MPV	1.28± 0.115†	1.18± 0.12	1.35 ± 0.16	1.304 ± 0.15	1.391 ± 0.1
RDW/PLT	0.09 ± 0.008	0.11 ± 0.01	0.15 ± 0.05*	0.07 ± 0.03	0.1 ± 0.05
SII	179.3 ± 25.07	290 ± 219.1	216.3 ± 62.62*	234.5 ± 58.62*	455.3 ± 37.56*

Values are presented as mean ± SE. Group 1:Control; Group 2: Benzene; Group 3: Benzene + Melatonin; Group 4: Benzene + Agomelatine; Group 5: Benzene + Melatonin + Luzindole. PLT: platelet count; MPV: mean platelet volume; PDW: platelet distribution width; PCT: plateletcrit; PLCR: platelet large cell ratio; PLCC: platelet large cell count; LYM: lymphocyte; WBC: white blood cell; RDW: red cell distribution width; SII: systemic immune-inflammation index. †P<0.05, ‡P<0.001 indicate significant differences compared with the Group 2; *P<0.05 indicate significant differences compared with the Group 3.

### Micronucleus Assay

Benzene exposure produced marked changes in erythropoietic and genotoxicity-related endpoints compared with the control group. The benzene group showed a reduction in %PCE and PCE/NCE ratio, together with an increase in %NCE, %MN-PCE, and %MN-NCE. Treatment with melatonin improved the erythropoietic indices and reduced micronucleus formation compared with the benzene-only group. Agomelatine also improved these parameters, with a pattern generally comparable to melatonin for several micronucleus-related endpoints. In the group 5, the improvement in %PCE, %NCE, %MN-PCE, %MN-NCE, and PCE/NCE ratio was less pronounced than in the melatonin-treated group. These results indicate that luzindole partially modified the protective pattern observed with melatonin treatment. All statistical comparisons and significance levels are presented in Figure-1 and Figure-2.

### Differential Leukocyte Counts

As shown in Table1, benzene exposure altered differential leukocyte counts compared with the control group. The benzene-only group showed lower WBC, LYM, and MON values, with changes also observed in NEU, EOS, and BAS counts. Melatonin treatment partially improved several leukocyte parameters compared with the benzene group. Agomelatine treatment also showed partial restoration of leukocyte distribution. Benzene + melatonin + luzindole exhibited distinct responses compared with melatonin alone, implying that receptor blockade may modify selected leukocyte-related effects of melatonin. These observations, however, need to be taken cautiously, because not all leukocyte parameters changed in the same direction. Specific values and significance annotations are found in Table1.

### Differential Leukocyte Ratios

The effects of benzene, melatonin, agomelatine, and luzindole on leukocyte ratios are presented in Figure-3. Benzene exposure altered LYM/MON, NEU/LYM, and NEU/MON ratios compared with the control group. Melatonin and agomelatine improved selected leukocyte ratios compared with the benzene-only group, particularly LYM/MON and NEU/MON. The NEU/LYM ratio showed a less consistent treatment-related pattern than the other leukocyte ratios. In the group 5, selected ratio changes differed from the melatonin-treated group, indicating partial modification of melatonin-associated effects. Statistical comparisons are provided in Figure-3.

### Platelet Count and Indices

Table2 and Figure-4 show platelet count and associated indices. Benzene exposure was associated with changes in several platelet and inflammation-related parameters compared with the control group, such as PLT, MPV, PDW, PCT, MPV/PLT, PLT/LYM, WBC/MPV, RDW/PLT, SII, %PLCR, and PLCC. Melatonin and agomelatine treatment led to variable changes in the parameters affected by benzene. However, for the group 5, some platelet-related values differed from those observed with melatonin alone. Thus, these platelet findings may better be interpreted parameter by parameter rather than as a single treatment response. Detailed numerical values and significance levels are presented in Table2 and Figure-4.

### Antioxidant Enzymes and Oxidative Stress Biomarkers

Figure-5 presents Activin A and Follistatin levels across the experimental groups. Follistatin did not show significant differences among groups. Activin A decreased in the benzene group compared with the control group. Melatonin and agomelatine showed different patterns of change in Activin A, while the benzene + melatonin + luzindole group showed a higher Activin A level compared with the benzene group. Because Activin A and Follistatin did not change in a parallel or consistent manner, the Activin A/Follistatin axis should be interpreted cautiously. Figure-6 presents oxidative stress and antioxidant-related biomarkers. Benzene exposure increased MDA compared with the control group, while CAT activity was reduced. Melatonin and agomelatine reduced MDA and improved CAT activity compared with the benzene-only group. SOD did not show a clear significant difference among the experimental groups. The 8-OHdG pattern did not follow the expected benzene-induced increase and therefore requires cautious interpretation. Full statistical comparisons are presented in Figure-6.

## Discussion

The present study evaluated the effects of melatonin, agomelatine, and luzindole on benzene-induced hematological, genotoxic, oxidative, and inflammatory alterations in rats. The findings showed that repeated benzene exposure produced clear disturbances in erythropoietic indices, micronucleus formation, leukocyte distribution, platelet-related parameters, oxidative stress markers, and Activin A levels. Treatment with melatonin and agomelatine attenuated several of these alterations, whereas luzindole partially modified selected protective responses. These findings suggest that melatoninergic modulation may contribute to protection against benzene-induced pre-leukemic alterations, although the results should be interpreted cautiously because the study design does not permit definitive separation of receptor-dependent and receptor-independent mechanisms.

Benzene exposure caused marked disruption of erythropoietic and genotoxicity-related endpoints, as reflected by reduced %PCE and PCE/NCE ratio together with increased %MN-PCE and %MN-NCE. These findings are consistent with the known hematotoxic and genotoxic effects of benzene metabolites on bone marrow cells [1, 2, 7].

Benzene-induced oxidative stress, chromosomal injury, and interference with hematopoietic cell maturation may contribute to impaired erythropoiesis and increased micronucleus formation [3, 5, 6]. Melatonin reduced micronucleus frequency and improved erythropoietic indices, suggesting a protective effect against benzene-induced cytogenetic injury. This effect may be related to its antioxidant and anti-inflammatory properties, as well as its ability to preserve cellular integrity under oxidative stress [10, 11, 15, 16]. Agomelatine also improved several micronucleus-related endpoints, which may reflect melatonergic receptor activity and/or additional cytoprotective effects [17, 22]. However, because agomelatine was not combined with luzindole in the present design, conclusions regarding direct receptor blockade of agomelatine cannot be made [22].

The leukocyte findings also indicate that benzene exposure disturbed immune cell distribution. Alterations in WBC, LYM, MON, NEU, EOS, and BAS counts suggest inflammatory and hematopoietic imbalance after benzene administration. Melatonin and agomelatine partially restored several leukocyte parameters, indicating possible immunomodulatory and hematoprotective effects [23, 24]. These effects may be associated with reduced inflammatory signaling and oxidative injury in the hematopoietic environment [10, 23, 24]. Nevertheless, not all leukocyte variables changed in the same direction, and some parameters showed only partial improvement. Therefore, the leukocyte data should be interpreted as evidence of partial immune modulation rather than complete normalization of benzene-induced hematological injury.

The alterations in leukocyte proportions reflect systemic inflammatory disturbance in benzene-exposed rats. Agomelatine and melatonin increased selected ratios, especially LYM/MON and NEU/MON, whereas NEU/LYM had a variable response to therapy. Indicative of melatoninergic effect on inflammatory regulation, these data indicated mixed effect of melatonin on inflammatory response but the pattern did not vary among all derived ratios [23, 25].

The partial modification in the melatonin + luzindole section indicates that at least some effects can relate to melatonin receptor-dependent mechanisms. Nevertheless, an effect of receptor-independent antioxidant and anti-inflammatory action of melatonin is possible [13, 15, 16].

Examination of platelets identified benzene exposure was associated with several platelet indices and inflammation parameters. Melatonin and agomelatine changed some platelet defects, but the degree and nature of these effects differed between PLT, MPV, PDW, PCT, PLCR, PLCC, PLT/LYM, and SII. Such observations indicate that the platelet response of this model is complicated and could be the product of a combination of bone marrow stress, systemic inflammation, oxidogenic insult and compensatory haematopoietic effect [7, 8]. So, the platelet observations should not be taken as a standard platelet-protection effect. Instead, they suggest that melatoninergic intervention may modulate select platelet cell responses under benzene-induced haematotoxic background. Under oxidative stress conditions with benzene-induced MDA and CAT, a larger increase and a weaker CAT effect were observed [7–9].

Melatonin and agomelatine reduced MDA levels and increased CAT activity, indicating partial restoration of the oxidant-antioxidant balance. These findings are consistent with the reported antioxidant properties of melatonin and the cytoprotective effects of agomelatine in oxidative stress models [15, 16, 22].

SOD levels did not differ clearly among groups, and 8-OHdG did not show the expected increase in the benzene group. This unexpected pattern may reflect the timing of sampling, tissue compartment, assay sensitivity, compensatory DNA repair, or differences between circulating and tissue-level DNA damage. Therefore, the 8-OHdG and SOD findings should be interpreted cautiously and should not be considered definitive evidence of oxidative DNA damage or antioxidant recovery [22, 28].

These findings should therefore be considered supportive rather than primary evidence for involvement of this pathway. The luzindole group provides only partial mechanistic information. Luzindole attenuated selected protective effects of melatonin, suggesting that melatonin receptor-dependent pathways may contribute to some responses [13]. However, the absence of a benzene plus agomelatine plus luzindole group prevents assessment of whether agomelatine-mediated effects were receptor dependent in this model. Moreover, melatonin may act through both receptor-dependent and receptor-independent mechanisms, including direct free-radical scavenging and modulation of inflammatory signaling [13, 15, 16].

Thus, the present findings support possible involvement of melatonin receptor-associated mechanisms but do not demonstrate that all protective effects were receptor mediated. Overall, melatonin and agomelatine attenuated several benzene-induced hematological, genotoxic, platelet-related, and oxidative alterations in rats. Further studies incorporating additional receptor-blockade groups, tissue-level assessment of oxidative DNA damage, molecular pathway analyses, and longer follow-up are warranted.

## Conclusion

Melatonin and agomelatine reduced several benzene-induced pre-leukemic changes in rats, including disturbances in hematological parameters, micronucleus formation, platelet-related indices, and oxidative stress balance. The improvement in erythropoietic markers, the lower MN-PCE and MN-NCE frequencies, and the partial recovery of MDA and CAT levels indicate a protective effect against benzene-related hematotoxic, genotoxic, and oxidative injury. Luzindole altered some of the effects observed with melatonin, which suggests that melatonin receptor-related pathways may be involved in part of the response. However, the absence of an agomelatine + luzindole group, together with the inconsistent or limited changes in Activin A, Follistatin, SOD, and 8-OHdG, means that the mechanistic explanation should be interpreted with caution. Further studies are required to clarify the relative contribution of receptor-mediated and non-receptor antioxidant and anti-inflammatory mechanisms in benzene-induced pre-leukemic models.

## Conflict of Interest

The authors declare no conflict of interest.

## AI Disclosure Statement

The authors used an artificial intelligence-based language model (chatGPT) only for language editing, grammar improvement, formatting guidance, and refinement of manuscript clarity during revision. The tool was not used for data generation, statistical analysis, interpretation of results, or formulation of scientific conclusions. All content was critically reviewed, verified, and approved by the authors, who take full responsibility for the integrity and accuracy of the manuscript.
